# Phosphodiesterase PDE4D Is Decreased in Frontal Cortex of Aged Rats and Positively Correlated With Working Memory Performance and Inversely Correlated With PKA Phosphorylation of Tau

**DOI:** 10.3389/fnagi.2020.576723

**Published:** 2020-10-28

**Authors:** Shannon N. Leslie, Dibyadeep Datta, Kyle R. Christensen, Christopher H. van Dyck, Amy F. T. Arnsten, Angus C. Nairn

**Affiliations:** ^1^Interdepartmental Neuroscience Program, Yale University, School of Medicine, New Haven, CT, United States; ^2^Department of Psychiatry, Yale University, School of Medicine, New Haven, CT, United States; ^3^Department of Neuroscience, Yale University, School of Medicine, New Haven, CT, United States; ^4^Department of Neurology, Yale University, School of Medicine, New Haven, CT, United States

**Keywords:** tau, working memory, PDE4D, aging, Alzheimer’s disease

## Abstract

Age is the largest risk factor for Alzheimer’s disease (AD) and contributes to cognitive impairment in otherwise healthy individuals. Thus, it is critical that we better understand the risk aging presents to vulnerable regions of the brain and carefully design therapeutics to address those effects. In this study we examined age-related changes in cAMP-regulatory protein, phosphodiesterase 4D (PDE4D). Inhibition of PDE4D is currently under investigation as a therapeutic target for AD based on memory-enhancing effects in rodent hippocampus. Therefore, it is important to understand the role of PDE4D in brain regions particularly vulnerable to disease such as the frontal association cortex (FC), where cAMP signaling can impair working memory via opening of potassium channels. We found that PDE4D protein level was decreased in the FC of both moderately and extremely aged rats, and that PDE4D level was correlated with performance on a FC-dependent working memory task. In extremely aged rats, PDE4D was also inversely correlated with levels of phosphorylated tau at serine 214 (S214), a site phosphorylated by protein kinase A. *In vitro* studies of the PDE4D inhibitor, GEBR-7b, further illustrated that inhibition of PDE4D activity enhanced phosphorylation of tau. pS214-tau phosphorylation is associated with early AD tau pathology, promotes tau dissociation from microtubules and primes subsequent tau hyperphosphorylation at other critical AD-related sites. Age-related loss of PDE4D may thus contribute to the specific vulnerability of the FC to degeneration in AD, and play a critical role in normal cAMP regulation, cautioning against the use of pan-PDE4D inhibitors as therapeutics.

## Introduction

Age-related cognitive decline is a growing public health concern with the number of individuals over the age of 65 expected to reach 1.5 billion worldwide by 2050 (United Nations, 2020). After the age of 65 an individual’s risk of developing Alzheimer’s disease (AD), a severe dementia, doubles every 5 years (Alzheimer’s Association, 2020). Even “healthy” aging is associated with decrements in memory encoding and working memory (Swerdlow, 2011). In both cases, specific circuits of the brain are preferentially affected. The cortico-cortical circuits of the association cortex and highly connected circuits of the hippocampus (Morrison and Hof, 1997) are particularly vulnerable to dysfunction and degeneration. Better understanding of how age makes these circuits vulnerable to dysfunction and AD pathogenesis is critical to advancing therapeutics.

Important insights into why cortico-cortical circuits of the association cortex are particularly vulnerable may arise from fundamental differences in the molecular regulation of newly evolved circuits (Arnsten et al., 2012). Cortico-cortical synapses in the dorsolateral prefrontal cortex (dlPFC) utilize recurrent excitation to facilitate delay-cell firing that is critical to working memory (Goldman-Rakic, 1995). cAMP-protein kinase A (PKA) signaling is one important mechanism by which PFC neurons regulate their firing. Synapses in the dlPFC underlying working memory are enriched in cAMP-PKA machinery including proteins that stimulate production and degradation of cAMP (Paspalas et al., 2013). Elevated levels of cAMP have repeatedly been shown to impair PFC-dependent function (Taylor et al., 1999; Ramos et al., 2003; Runyan and Dash, 2005). Therefore, it is critical that the PFC maintains appropriate regulation of cAMP levels.

cAMP phosphodiesterases (PDEs) play a critical role in regulating cAMP levels. There are 11 different families of PDEs (PDE1–11) with numerous isoforms in each family (Kelly, 2018). PDE4s are of particular interest given their relatively high expression in brain and reported role in cognition (Richter et al., 2013). Specifically, the PDE4D subfamily has been proposed as a therapeutic target for AD given reported cognitive-enhancing effects of PDE4D inhibitors that are able to avoid negative gastro-intestinal side-effects of other PDE4 inhibitors (Bruno et al., 2011; Tibbo et al., 2019). However, much of this research is based on studies of cognition linked to the hippocampus (Tibbo et al., 2019) and results may not be applicable across brain regions. Notably, elevated PDE4D expression uniquely distinguishes the PFC from other brain regions (Carlyle et al., 2017) and thus, likely plays an important role in PFC function. Therefore, it is critical to understand how PDE4D functions in the PFC, specifically, in order to assess the safety and utility of PDE4D inhibitors for cognitive enhancement.

Previous work has shown that PDE4s (PDE4A5 and PDE4D) are lost from cortico-cortical synapses and dendrites, respectively, with advanced age in primates (Carlyle et al., 2014; Datta, Leslie, Nairn and Arnsten, unpublished). Elevated cAMP-PKA signaling in aging primates results in decreased delay-related firing during a working memory task through activation of hyperpolarization-activated cyclic-nucleotide gated (HCN) cation and KCNQ potassium channels (Wang et al., 2011). Additionally, dysregulated PDE4 is associated with elevated tau phosphorylation at serine 214 (pS214-tau) (Carlyle et al., 2014), a site that detaches tau from microtubules (Illenberger et al., 1998) and primes subsequent phosphorylation by GSK3β (Liu et al., 2004). Neurofibrillary tangles composed of hyperphosphorylated tau are a key hallmark of AD pathology and are rich in pS214-tau (Augustinack et al., 2002). The accumulation of tau pathology is a complex process likely involving numerous modification sites but pS214-tau provides one metric to better understand pathological vulnerability. Thus, age-related loss of PDE4 regulation of cAMP may contribute to cortical vulnerability and PDE4 inhibitors may be detrimental to PFC-dependent functions.

In this study we examined age-related changes in PDE4D protein levels in the frontal cortex (FC) of rats, the cortex anterior to the genu of the corpus callosum. We found a decrease in PDE4D levels at the most advanced ages and a more moderate, but still significant, decrement at less advanced ages. In addition, PDE4D levels correlated with performance on a working memory task. Levels of pS214-tau were relatively low in wild-type rats, as expected; nonetheless, in the most advanced aged cohort there was a significant increase that negatively correlated with PDE4D levels. This association between PDE4D activity and pS214-tau was confirmed *in vitro* in primary neuron cultures, utilizing GEBR-7b a PDE4D specific inhibitor. Thus, PDE4D was decreased in the FC of aged rats where it was positively correlated with cognitive performance and negatively correlated with pS214-tau phosphorylation.

## Materials and Methods

All experiments were conducted in accordance with the guidelines of Yale University Institutional Animal Care and Use Committee, and Public Health Service requirements for animal use as described in the Guide for the Care and Use of Laboratory Animals. Sprague Dawley rats were cared for under the guidelines of the National Institutes of Health and Yale IACUC. Yale University is accredited by the American Association for Accreditation of Laboratory Animal Care (AAALAC).

### Subjects

All rats used in this study were Sprague Dawley males. Rearing aged rats is a difficult process and many animals do not reach even 25 months of age. As such, aged rats are pre-ordered a year in advanced from Harlan/Envigo and are all males. Initially, 25-month-old rats were trained for cognitive characterization but too few animals survived to training and thus a second, younger, cohort was supplemented (details of ordering and age in supplement). The extreme aged animals came from a variety of cohorts where only a few animals (*N* = 3) survived to the target age of 30 months; however, we collected a full cohort (*N* = 10) with an average age of 27.8 months. Tissue from this extreme aged cohort was also analyzed in Datta et al., 2020. These fragile animals were not cognitively tested.

### Behavioral Characterization

The delayed-alternation *T*-maze was chosen for assessment of FC spatial working memory (Zahrt et al., 1997). Details of animal handling and training are in the supplement. Briefly, once animals were sufficiently trained on the task, they were tested on variable delays beginning with 0, 5, 10, and 15 s delays that were randomly distributed through the training session. The 0 s delay includes the time it takes to move the animal from the arm of the *T*-maze back to the beginning of the maze and remove the starting gate (∼3 s), so it does encompass some working memory but is used as a baseline. Animals were tested on this paradigm for 20 days. If animals began performing over 90% correct for two consecutive days, they were extended to 0, 10, 20, 30 s delays to prevent over-training. Four animals met this criterion; three young animals and one aged animal. Thus, cognitive data is presented as the average correct alternations across all test days on which the specific animal received that delay. Every animal received 60 trials of the 0 and 10 s delays; however, only the 10 s delay requires significant working memory capability. All behavioral data are from a cohort of twelve young animals and eight aged animals that completed all 20 days of testing. Two aged animals did not survive for the full testing period and are included in the biochemical but not the cognitive analysis.

### Tissue Preparation

Animals were euthanized by rapid decapitation following brief anesthesia with isoflurane. Brains were removed as quickly as possible. The frontal cortex block (FC) was dissected with a razor blade; a block of tissue sufficient for biochemical analysis was required, and thus all the cortical tissue anterior to the genu of the corpus callosum (i.e., cut at approximately AP 1.9), excluding the olfactory bulb was taken. The tissue block was split between hemispheres, and immediately frozen on dry ice. The entire process took approximately 5 min. In the case of triton-soluble lysate from cognitively characterized animals, lysate was immediately prepared. Tissue was stored at −80°C until analysis. Triton-soluble lysate was prepared as previously described including a 5 min, 15,000 × *g*, centrifugation at room temperature (Datta et al., 2020). Total lysate was collected after rotor homogenization but prior to any centrifugation for the extreme aged cohort. Total lysate for the cognitively characterized cohort was collected at the beginning of a synaptosomal preparation. Synaptosomal samples were rotor homogenized in a sucrose buffer with phosSTOP phosphatase inhibitor, and cOmplete mini protease inhibitor. In all cases samples were boiled for 5 min at 100°C in SDS-loading buffer with DTT prior to analysis via western blot.

### Western Blotting

Samples were run on 4–20% Tris–glycine SDS-PAGE gels and transferred onto nitrocellulose membranes. Membranes were blocked with 5% milk and incubated overnight in primary antibody. Antibodies used for this study included Total Tau46 (CST 4019, 1:1000), phospho-Tau S214 (Abcam ab4846, 1:1000), GAPDH (Millipore CB1001-500, 1:10,000), and PDE4D (Millipore ABS22, 1:1000). Membranes were washed three times in PBS-T and incubated with a species-specific secondary antibody. Blots were developed utilizing a LI-COR Odyssey scanner. Further details are included in the supplement.

### Primary Neuron Cultures

The protocol for primary cortical neuron culture preparation was as described in detail in the supplement. Briefly, E19 rat embryos were collected and brains were extracted. Meninges were removed and cortical regions were dissected. Neurons were dissociated with needles of increasing gage. Cells were grown with neurobasal in poly-D-lysine coated six well plates.

Experiments were performed at 13 days-*in vitro* (DIV). GEBR-7b (Millipore 524748) and Forskolin (FSK) (Fisher 109910) were dissolved in DMSO. The stocks were diluted in culture media. Cells were incubated in 100 μM GEBR-7b or an equivalent volume of DMSO for 10 min followed by 600 nM FSK (Carlyle et al., 2014) or DMSO as specified for an additional 10 min. Following incubation, cells were rinsed with ice-cold PBS, then lysed via sonication in warmed 1% SDS buffer with phosphatase and protease inhibitors.

### Immunohistochemistry

For PDE4D and pS214-tau immunolabeling, all male three young (3 month) and three aged (27 to 29-month-old) Sprague-Dawley rats were used. Animals were anesthetized with Nembutal (50 mg/mL, i.p.) and perfused transcardially with 4% paraformaldehyde (PFA) in 0.1 M phosphate buffer (PB; pH7.4). After perfusion, brains were removed from the skull and immersed in 4% PFA overnight at 4°C. Coronal 60 μm-thick sections were then cut on a Vibratome (Leica V1000) and collected in 0.1 ml PB. Sections of the medial PFC (mPFC) were transferred for 1 h to Tris–buffered saline (TBS) containing 5% bovine serum albumin, plus 0.05% Triton X-100 to block non-specific reactivity, and incubated in rabbit PDE4D antibody (SAB4502128; Millipore Sigma Aldrich) at 1:200 dilution, or rabbit pS214-tau antibody (ab4846; Abcam) at 1:100 dilution, in TBS for 48 h at 4°C. The tissue sections were incubated in goat anti-rabbit biotinylated antibody (Vector Laboratories) at 1:300 in TBS for 2 h, and developed using the Elite ABC kit (Vector Laboratories) and diaminobenzidine (DAB) as a chromogen. Omission of the primary antibody eliminated all labeling. Sections were mounted on microscope slides and examined and photographed under an Axiophot microscope equipped with Axiocam camera (Zeiss).

### Statistical Analysis

All statistical analyses were performed in Graphpad Prism. Protein levels were normalized for loading (details in supplement). Values were normalized to the average value of the young animals or control wells within each blot. The distribution of values was tested utilizing a D’Agostino and Pearson normality test and an *F*-test was used to compare variances. The appropriate statistical test to compare means was chosen based on these results and is specified in the figure legends. In all bar graphs the standard error of the mean (SEM) is presented to reflect variance within the groups. Correlations were computed utilizing a linear regression after confirming normal distributions.

### Graphics

Graphs were made on Graphpad Prism. Cartoon images were created with BioRender.com.

## Results

### PDE4D Protein Level Is Decreased in Rat FC With Age and Positively Correlates With Cognitive Performance

**FIGURE 1:**
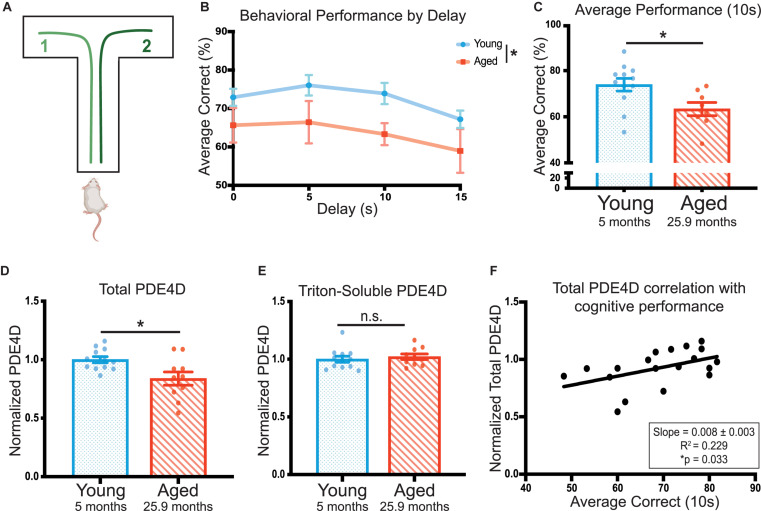
PDE4D in rat frontal cortex is decreased with age and is correlated with working memory performance. **(A)** A schematic of the delayed-alternation *T*-maze task used to characterize cognitive performance in rats. **(B)** Animal performance on the alternating *T*-maze across delay times. Performance is represented as percent correct for a given delay time across all days the animals were tested for that delay. Young animals (*N* = 12) are shown in blue and aged animals (*N* = 8) are shown in red. The standard error of the mean (SEM) is plotted for each dataset. Groups were compared utilizing a two-way ANOVA and there was a significant effect of age [*F*(1,18) = 4.479, *p = 0.0485]. **(C)** The average performance of each animal across 10 s delay (20 days, 60 trials) with SEM. Average performances were compared with an unpaired *t*-test between young (blue, *N* = 12) and aged (red, *N* = 8) animals (*p = 0.0195). **(D)** Total PDE4D normalized by GAPDH was compared between young (blue, *N* = 10) and aged (red, *N* = 10) by a Welch’s *t*-test (*p = 0.0362). **(E)** Triton-soluble PDE4D normalized by GAPDH was compared between young (blue, *N* = 10) and aged (red, *N* = 10) animals by a Mann-Whitney test (*p* = 0.4176). Two aged animals were included in the lysate analysis despite being unable to complete the task. Bar graph represents mean and SEM. The average age of each cohort is displayed beneath graphs **(C–E)**. **(F)** Normalized total PDE4D levels are plotted by the animal’s average correct performance on the 10 s delay. The relationship between the two measures was fit by a linear regression utilizing a Pearson correlation to determine significance (*p = 0.033). The correlation coefficient and slope with standard error are presented in the box in the lower right corner of the graph.

Cognitive ability was tested over a 20 day period using the delayed alternation spatial working memory task in a *T*-maze (Figure 1A). Age significantly impacted rat performance on the task [*F*(1,18) = 4.48, *p = 0.049] (Figure 1B). Aged rats (25 and 28 months) showed significantly impaired average performance on the 10 s delay compared to young animals (5 months; *p = 0.020) (Figure 1C). Given each animal received 60 trials at this delay, the 10 s delay was utilized as the metric of cognitive performance for correlations with biochemical measures.

To test whether impaired cognitive performance might be related to cAMP regulation, we assayed levels of PDE4D in FC. ImmunoEM of PDE4D expression in the primate PFC has shown that it is closely associated with dendritic microtubules, membranes of the smooth endoplasmic reticulum, near the post-synaptic density (PSD) in spines, and extensively expressed in the nucleus (Datta, Leslie, Nairn and Arnsten, unpublished). Furthermore, analysis of PDE4A5 revealed localization specific changes (Carlyle et al., 2014). Thus, we utilized two lysis conditions and multiple fractions in our assays of PDE4D levels. In the cognitively characterized cohort, we analyzed a total of three fractions: 1) total crude lysate from a synaptosome preparation, 2) a crude synaptosomal fraction, which captures pre- and post-synaptic compartments, and 3) a triton-soluble fraction which contains proteins soluble in 1% Triton X-100 but cleared by centrifugation. The total crude lysate preparations showed a significant decrease in PDE4D expression in the cognitively assessed aged animals compared to the young controls (^∗^*p* = 0.023) (Figure 1D, Supplementary Figure 1A). There was no difference between young and aged animals in PDE4D in synaptosomal (Supplementary Figures 1C,D) or triton-soluble lysates (Figure 1E, Supplementary Figure 1B). Importantly, total PDE4D levels detected in the crude lysate preparation positively correlated with cognitive performance (^∗^*p* = 0.033) (Figure 1F).

### Advancing Age Exacerbates Loss of PDE4D Decrease in the Rat FC

Given the subtle but significant differences we observed in PDE4D protein levels in the cognitively characterized cohort, we examined this in a more advanced aged cohort. However, cognitive characterization requires months of training and testing, and we were unable to maintain a sufficient number of cognitively assessed animals into extreme age. Thus, we collected a cohort of older aged rats without cognitive testing and compared them to young controls. This “extreme aged” cohort consisted of aged animals (24.5–30 months of age) and young animals (3.5 months of age). These aged animals were significantly older than those in the cognitively characterized cohort (Supplementary Figure 2A,^∗^*p* = 0.012).

**FIGURE 2:**
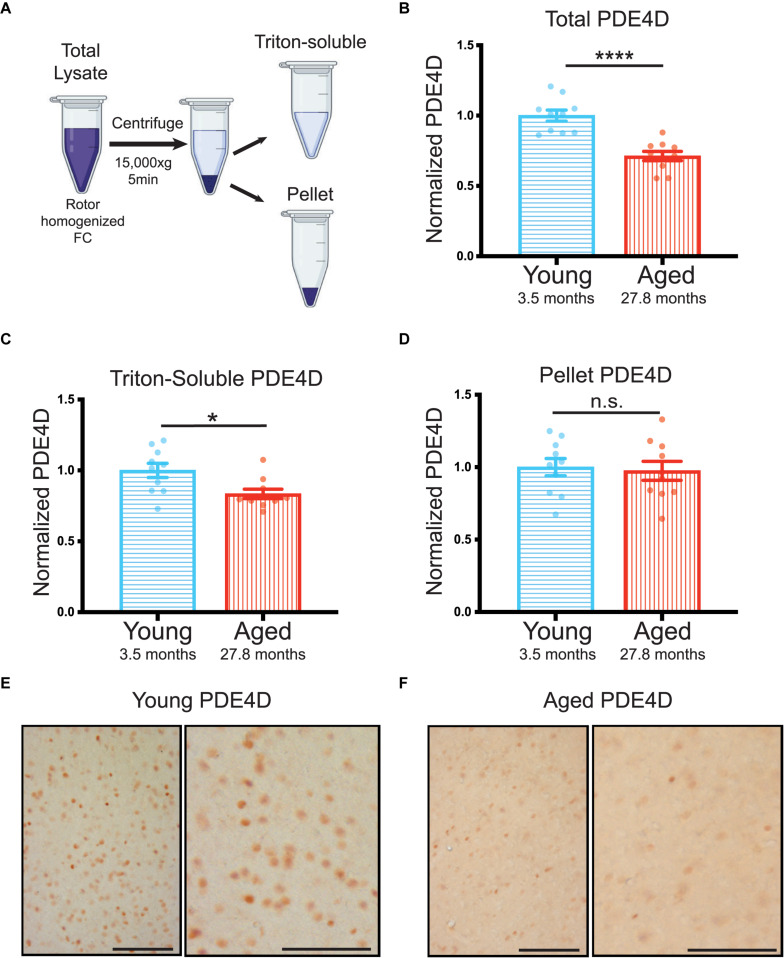
Advanced age highlights loss of PDE4D particularly from total lysate in rat FC. **(A)** Schematic demonstrating the process from crude total lysate to triton-soluble lysate and pellet. **(B)** Levels of total PDE4D normalized to GAPDH were compared between young (blue, *N* = 10) and aged (red, *N* = 10) animals. An unpaired *t*-test showed a significant difference between the two age groups (****p < 0.0001). Bar graph represents mean and SEM **(C)** Quantification of triton-soluble PDE4D normalized to GAPDH in an extreme age cohort of young and aged animals. Young animals in blue (*N* = 10) were compared to aged animals in red (*N* = 10) via a Mann-Whitney test (*p = 0.0232). Bar graph represents mean and SEM **(D)** PDE4D was detected in the pellet created by the centrifugation step between total lysate and triton-soluble lysate. Comparison of PDE4D in the pellet between young and animals was conducted via an unpaired *t*-test and revealed no difference (*p* = 0.7026). Variation within groups is noted by the SEM depicted with each bar graph. The average age of each cohort is displayed beneath graphs **(B–D)**. **(E)** Expression of PDE4D in young rat mPFC layer II/III. Pyramidal neurons in layer II/III were immunolabeled against PDE4D, especially with staining being found in the pyramidal somata and delicate labeling in the neuropil visualized in the higher-magnification panel (right). **(F)** Expression of PDE4D in aged rat mPFC layer II/III. There was a marked decrement in the density of PDE4D-immunopositive neurons in aged rat mPFC layer II/III observed in the higher-magnification panel (right). Scale bar, 100 μm.

For this extreme aged cohort, we collected total lysate prior to triton-soluble preparation by centrifugation. We analyzed both total and triton-soluble lysates, as well as the pellet, that is the only material difference between the two lysates (Figure 2A), in order to better understand the discrepancies between total and triton-soluble lysates observed in the cognitively characterized cohort. The extreme aged cohort showed a significant decrease in PDE4D protein levels in both the total crude lysate (^****^p < 0.0001) (Figure 2B, Supplementary Figure 2B), and the triton-soluble lysate (^∗^*p* = 0.023) (Figure 2C, Supplementary Figure 2C). Notably, the decrease in PDE4D levels in the extreme aged cohort was larger (−0.29 ± 0.05) than the cognitively characterized cohort (−0.16 ± 0.06), indicating that more advanced age can increase the magnitude of PDE4D loss.

In both cohorts there was a notable difference between triton-soluble and total lysates. To better understand the differences between the total crude lysate and the triton soluble lysate, we analyzed PDE4D levels in the pellet that resulted from the centrifugation step between the two lysates (Figure 2A). PDE4D was evident in an SDS/DTT soluble preparation of the pellet from the extreme age cohort (Supplementary Figure 2D); however, there was no significant difference between young and aged animals (Figure 2D). It is notable that the SEM is much larger in the pellet than in total and triton-soluble lysates (Figure 2D vs. Supplementary Figures 2B,C). Thus, variable PDE4D levels in the pellet may obscure analysis of triton-soluble lysates.

These biochemical results were confirmed by immunohistochemistry showing decreases in PDE4D immunolabeling in medial (mPFC) layer II/III in aged rats (Figure 2E,F). In young rats, PDE4D staining was observed in the perisomatic compartment and in delicate processes in the neuropil (Figure 2E). In contrast, in extremely aged rats, a marked decrement was observed in the density of PDE4D-immunopositive neurons in mPFC layer II/III (Figure 2F). The decrease in the density of PDE4D-immunopositive cells with advancing age is observed in low magnification micrographs across cortical layers in rat mPFC (Supplementary Figures 3A,B). The aging immunohistochemistry PDE4D data are consistent with biochemical measures capturing a decrease in PDE4D protein levels in the extreme age cohort.

### Levels of PDE4D Inversely Correlate With pS214-Tau in Rat FC

**FIGURE 3:**
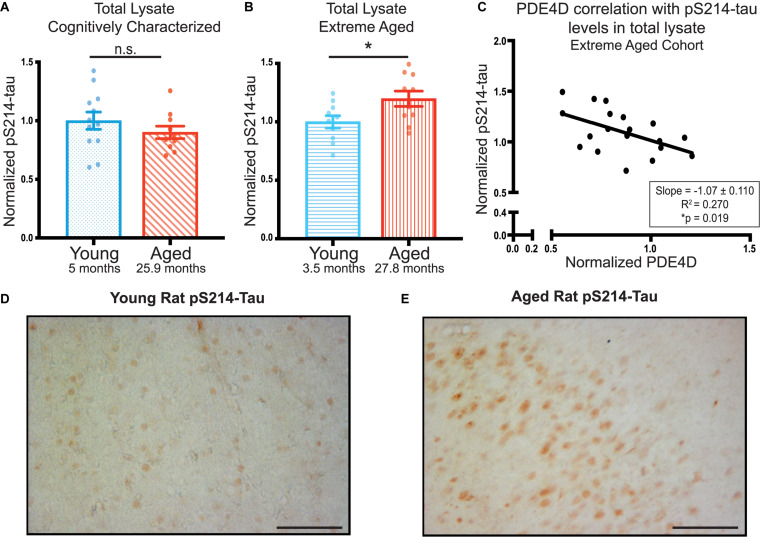
PDE4D inversely correlates with elevated pS214-tau in FC in an extreme aged cohort of rats. **(A)** Levels of pS214-tau normalized to total tau in total lysate were compared between young (blue dots) and aged (red diagonal lines) animals in the cognitively characterized cohort. No significant difference between young and aged animals was found using an unpaired *t*-test (*p* = 0.3069). **(B)** Levels of normalized pS214-tau were compared between young (blue horizontal lines) and aged (red vertical lines) animals in the extreme age cohort. A significant difference was detected by an unpaired *t*-test (*p = 0.0308). Bar graph represents mean and SEM. **(C)** Levels of normalized pS214-tau were plotted against levels of PDE4D in the extreme age cohort. The relationship between the two markers was fit by linear regression utilizing Pearson Correlation. The slope and correlation coefficient are shown in the box in the bottom right of the graph. The slope was significantly non-zero (*p = 0.019). The average age of each cohort is displayed beneath graphs **(A,B)**. **(D)** Expression of pS214-tau in young rat mPFC layer II/III. Immunolabeling was virtually absent within neurons and the neuropil. **(E)** Expression of pS214-tau in aged rat mPFC layer II/III. There was a marked elevation in the density and intensity of pS214-tau immunopositive neurons. Scale bar, 100 μm.

In order to assess the possible role of PDE4D loss on neuronal integrity and its potential role in increasing AD pathology risk, we examined phosphorylation of tau at S214, a phosphorylation site that is evident in early AD pathology (Augustinack et al., 2002). We did not find any significant age-dependent difference in pS214-tau levels in total lysate from the younger, cognitively characterized cohort (Figure 3A, Supplementary Figure 4A). However, we did find a significant increase in total pS214-tau levels in the aged animals in the extreme aged cohort (Figure 3B, Supplementary Figure 4B). Given these animals also had a larger loss of PDE4D, we examined the relationship between the two markers. There was a significant inverse correlation between PDE4D and pS214-tau in this cohort (Figure 3C), consistent with reduced regulation of cAMP signaling leading to increased PKA phosphorylation of tau.

Elevated levels of pS214-tau were confirmed in extreme aged rats using immunohistochemistry. In young rats, very little pS214-tau labeling was observed in layer II/III of mPFC (Figure 3D) or across cortical layers (Supplementary Figure 3C). In contrast, in aged rats, there was a marked elevation in the density and intensity of pS214-tau labeled neurons in prelimbic mPFC layer II/III (Figure 3E) and throughout multiple layers of the cortex (Supplementary Figure 3D), consistent with the quantitative biochemical data. The labeling for pS214-tau in aged rat mPFC layer II/III was concentrated in the soma, and along the early proximal segment of apical dendrites, with delicate punctate labeling in the neuropil, likely reflecting staining within subcompartments such as dendritic spines.

### Inhibition of PDE4D Increases PKA Phosphorylation of Tau at Ser214 in Cultured Cortical Neurons

**FIGURE 4:**
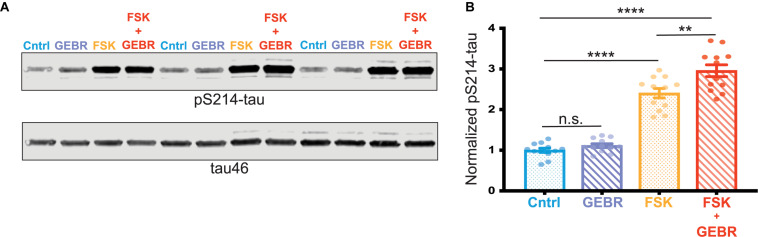
PDE4D inhibition exacerbates PKA phosphorylation of tau *in vitro*. **(A)** Representative blot of three replicates from one cortical neuron preparation. Control (Cntrl) samples, treated with DMSO, are labeled in blue, GEBR-7b samples are in purple, FSK samples are in orange, FSK + GEBR samples are in red. pS214-tau and total tau (tau46) were blotted on the same membrane so no loading control was used. **(B)** Quantification of normalized pS214-tau across three independent neuronal preparations, four experimental replicates per preparation (*N* = 12) are shown for each condition. Means for each group were compared using a Tukey’s multiple comparison test. The significance based on adjusted *p*-value is shown: Cntrl vs. GEBR *p* = 0.1429, Cntrl vs. FSK ****p < 0.0001, Cntrl vs. GEBR + FSK ****p < 0.001, FSK vs. GEBR + FSK **p = 0.0017. Bar graph represents mean and SEM.

To further assess a possible role of reduced PDE4D expression in the PKA phosphorylation of pS214-tau, we carried out studies *in vitro* utilizing primary rat cortical neurons. Neurons were treated with either DMSO (vehicle control), the PDE4D inhibitor (GEBR-7b) alone, the cAMP activator (forskolin [FSK]) alone, or both GEBR-7b and FSK. Activation of cAMP-PKA signaling with FSK significantly increased phosphorylation of tau at S214, and this effect was exacerbated by PDE4D inhibition with GEBR-7b (FSK vs. FSK + GEBR adjusted ^∗∗^*p*-value = 0.0017) (Figures 4A,B). These results are consistent with the hypothesis that age-related loss of PDE4D expression may contribute to tau phosphorylation by cAMP-PKA signaling.

## Discussion

These studies in rats demonstrated that PDE4D in FC was decreased with age. Levels of PDE4D correlated with spatial working memory and inversely correlated with levels of pS214-tau. These results suggest that age-related loss of PDE4D suppression of cAMP signaling may impair FC function and contribute to neuronal vulnerability. Our results caution that while PDE4D inhibitors may be effective at improving hippocampal-based memory, they may have deleterious effects in other aging and AD-related vulnerable regions of the brain.

Aged rats provide a unique window to study age and AD-related signaling changes as they possess elaborated cortico-cortical synapses and more human-like tau isoform expression compared to mice (Takuma et al., 2003), but are still a tractable model for molecular studies. However, the data presented here caution that the method of lysate preparation may affect PDE4D analysis, an important consideration for future studies.

### cAMP-PKA Signaling in the FC vs. Hippocampus

cAMP-PKA signaling is traditionally associated with enhanced plasticity and learning, e.g., in primitive organisms (Dudai et al., 1976; Tibbo et al., 2019), and in classic brain circuits such as the hippocampus (Kandel, 2012). However, cAMP signaling can impair the working memory functions of the newly evolved PFC. The differences in signaling mechanisms are consistent with the differing cognitive operations performed by these structures, where the hippocampus mediates longer term memory consolidation, while the PFC regulates working memory, where information is maintained and manipulated in short-term stores, i.e., generating a dynamically changing “mental sketch pad” (Arnsten et al., 2012). Extensive evidence shows that cAMP signaling enhances plasticity needed for long-term memory consolidation in hippocampal circuits (Abel et al., 1997; Kandel, 2012), and that PDE4 inhibition or knockdown in these circuits can strengthen hippocampal plasticity and improve memory consolidation (Havekes et al., 2016; Wimmer et al., 2020). Indeed, selective inhibition of PDE4D can improve mnemonic functions dependent on the hippocampus (Ricciarelli et al., 2017). In contrast, cAMP signaling in the PFC microcircuits that generate working memory can weaken synaptic connectivity and impair working memory by opening K^+^ channels near synaptic connections on spines [reviewed in Arnsten et al. (2012)]. Thus, activation of cAMP-PKA signaling, or inhibition of PDE4s, in primate PFC can reduce working memory-related neuronal firing (Wang et al., 2007). Studies of rodent mPFC also have demonstrated a negative effect of elevated cAMP on working memory function (Taylor et al., 1999; Runyan and Dash, 2005; Kobori et al., 2015). Thus, it is clear that FC and hippocampal circuits differ in their responses to cAMP-PKA signaling.

### PDE4D in Aging and AD

Given the importance of cAMP signaling to long-term and working memory, it is important to understand how age-related changes in PDE4D may contribute to age-related cognitive decline. With advancing age, there is loss of cAMP signaling in hippocampus, and increased cAMP signaling in PFC, that may harm both structures (Ramos et al., 2003). In the aging primate dlPFC, dysregulated cAMP-PKA-K^+^ signaling reduces the persistent firing needed for working memory (Wang et al., 2011). These data are consonant with the current results, where PDE4D expression in the FC correlated with working memory performance, and both PDE4D and cognitive performance were decreased with age. As PFC working memory abilities are expanded in humans, these regional differences in cAMP signaling should be considered when evaluating therapeutic agents targeting cAMP-PKA signaling in the brain.

Analysis of the human brain has also revealed decreased PDE4D transcript in the frontal cortex with advanced age (Lu et al., 2004), suggesting that the current results in rats may be directly relevant to humans. PDE4D transcript has also been shown to be decreased in AD in the hippocampus, which may be related to neuronal loss (McLachlan et al., 2007). However, mRNA is not always indicative of protein levels, and there is still debate over the changes in PDE4D activity with age (Tibbo et al., 2019). Nonetheless, the current results demonstrated that PDE4D protein levels are significantly decreased in the aged FC, supporting human transcriptome studies. Future research into changes across brain regions not only in total PDE4D protein but isoform and activity specific analyses will be important. Furthermore, understanding the drivers of age-related loss of PDE4D is critical. Previous work with the extreme aged cohort utilized in this study, revealed an age-related increase in complement signaling protein C1qA (Datta et al., 2020). Investigating the ways in which age-related signaling changes may contribute to loss of PDE4D may demonstrate new therapeutic avenues to alleviate age-related cognitive decline and potentially AD risk.

### PDE4D Inhibitors as Therapeutic Targets

Phosphodiesterase inhibitors have become an increasing target for therapeutics for potential cognitive enhancement, largely based on data from rodent hippocampus. The PDE4D family specifically has garnered recent interest given its abundance in the brain, and potential to avoid negative peripheral side-effects like nausea (Tibbo et al., 2019). PDE4D knock-out rats displayed enhanced hippocampal dependent memory function without causing overt gastro-intestinal issues that are common side effects of PDE4D inhibitors (Li et al., 2011). Additionally, systemic administration of the PDE4D selective inhibitor, GEBR-7b, elevated hippocampal levels of cAMP and also improved hippocampal dependent memory functions in rats (Bruno et al., 2011). However, PDE4D knockout did not enhance working-memory, consistent with the differing needs of hippocampal vs. PFC circuits (Li et al., 2011). The data from our current study illustrate that decreased levels of PDE4D in rat FC are associated with *impaired* rather than improved cognitive performance, cautioning that PDE4D inhibitors may impair higher cognition dependent on the frontal lobe. The confound of aging is important to bear in mind when investigating the connection between these markers; however, these data still suggest that further loss of PDE4D activity through use of an inhibitors could be detrimental rather than beneficial. Furthermore, loss of PDE4D activity may contribute to tau phosphorylation, as suggested by our *in vitro* results showing that GEBR-7b enhanced pS214-tau phosphorylation, and that PDE4D levels in FC inversely correlated with pS214-tau expression *in vivo*. PKA phosphorylation of tau at S214 can be particularly harmful in initiating tau pathology, as it causes tau to dissociate from microtubules and primes tau for hyperphosphorylation (Illenberger et al., 1998; Liu et al., 2004). Thus, PDE4D inhibition may be detrimental not only to PFC function but to PFC cellular integrity.

In summary, our results demonstrate that loss of PDE4D in cortical neurons can exacerbate PKA phosphorylation of tau and thus may contribute to increased risk of subsequent tau pathology. The results of this study indicate that PDE4D is decreased with age in the rat FC where it correlated with cognitive function and could potentially contribute to FC vulnerability to degeneration, raising caution about the use of PDE4D inhibitors for cognitive enhancement and AD treatment.

## Data Availability Statement

The raw data supporting the conclusions of this article will be made available by the authors, without undue reservation.

## Ethics Statement

The animal study was reviewed and approved by Yale Institutional Animal Care and Use Committee (IACUC) and Yale Veterinary Clinical Services (VCS).

## Author Contributions

SL and DD both contributed to the project’s hypothesis. SL designed, performed, and analyzed biochemical experiments and was one of the animal handlers for behavior. DD collected and analyzed immunohistochemistry experiments. SL and AA analyzed behavioral experiments. KC aided in analysis and interpretation of tau data and contributed to data that helped shape the project. CD contributed to the experimental design and revised the manuscript. AA and AN designed the experiments, supervised the study, and critically revised the manuscript. All authors read and approved the final manuscript.

## Conflict of Interest

AA and Yale University receive royalties from Shire/Takeda from the United States sales of Intuniv. They do not receive royalties from international sales or generic Intuniv. The remaining authors declare that the research was conducted in the absence of any commercial or financial relationships that could be construed as a potential conflict of interest.

## Supplementary Material

Supplementary Figure 1 PDE4D analysis in FC of cognitively characterized rats. **(A)** Representative blot of PDE4D in total sucrose lysate from the cognitively characterized cohort. All bands of PDE4D were utilized for quantification as illustrated by the quantified area outlined by the green rectangle. Lanes are labeled by their animal ID which is color-coded blue for young animals and red for aged animals. **(B)** Representative blot of PDE4D in the crude synaptosomal fraction from cognitively characterized animals. All bands of PDE4D were utilized for quantification as illustrated by the quantified area outlined by the green rectangle. **(C)** Crude synaptosomal PDE4D normalized by GAPDH was compared between young (blue, *N* = 10) and aged (red, *N* = 10) by an unpaired *t*-test (*p* = 0.6332). **(D)** Representative blot of PDE4D in triton-soluble lysate from the cognitively characterized cohort. Lanes are labeled by their animal ID which is color-coded blue for young animals and red for aged animals. All bands of PDE4D were utilized for quantification as illustrated by the quantified area outlined by the green rectangle.

Supplementary Figure 2 PDE4D analysis in FC of an extreme aged cohort of rats. **(A)** Ages of the two rat cohorts analyzed in this study. Young animals are shown in blue and aged animals are shown in red. The two groups are distinguished by the pattern of the box plot. Cognitively characterized animals are shown by dots for young animals and diagonal lines for aged. Extreme aged animals are represented by horizontal lines for young animals and vertical lines for aged animals. Sidak’s multiple comparison test was used to compare the age difference between cognitively characterized and extreme aged animals. The two aged cohorts significantly differed in age (^∗^*p* = 0.016). **(B)** Representative blot of PDE4D in total lysate from the extreme age cohort. All bands of PDE4D were utilized for quantification as illustrated by the quantified area outlined by the green rectangle. The same molecular weight region was analyzed in all blots **(B–D)**. Lanes are labeled by their animal ID which is color-coded blue for young animals and red for aged animals. **(C)** Representative blot of PDE4D in triton-soluble lysate from the extreme age cohort. Area of quantifications is highlighted by the green rectangle. Animals are labeled by their animal ID which is color-coded blue for young animals and red for aged animals. **(D)** Representative blot of PDE4D in the pellet resulting from the centrifugation of total lysate from the extreme age cohort. Area of quantifications is highlighted by the green rectangle. Lanes are labeled by their animal ID which is color-coded blue for young animals and red for aged animals.

Supplementary Figure 3 PDE4D and pS214-tau immunohistochemistry across cortical layers in rat prelimbic mPFC. **(A)** Immunolabeling for PDE4D in young rat mPFC. Immunopositive cells are distributed throughout the cortical neuropil across all layers. **(B)** Immunolabeling for PDE4D in aged rat mPFC, with sparser labeling across all cortical layers. **(C)** Expression of pS214-tau in young rat mPFC with very few immunopositive cells. **(D)** Increased density and intensity of pS214-tau labeling across cortical layers in aged rat mPFC. Approximate depth of cortical layers is demarcated in each micrograph. Scale bars, 100 μm.

Supplementary Figure 4 pS214-tau in rat FC. **(A)** Representative blot of pS214-tau and total tau in total FC lysates from a cognitively characterized cohort. Lanes are labeled by their animal ID which is color-coded blue for young animals and red for aged animals. **(B)** Representative blot of pS214-tau and total tau in total lysate from an extreme aged cohort. Lanes are labeled by their animal ID which is color-coded blue for young animals and red for aged animals.
