# Supplementary material for: Phosphodiesterase PDE4D Is Decreased in Frontal Cortex of Aged Rats and Positively Correlated With Working Memory Performance and Inversely Correlated With PKA Phosphorylation of Tau
Source: Front Aging Neurosci. 2020 Oct 28;12:576723. doi: 10.3389/fnagi.2020.576723 (PMC7655962; doi:10.3389/fnagi.2020.576723)
Supplement: Supplementary file 1 [file Data_Sheet_1.docx]

**Supplemental Methods**

**Animal Handling**

All animals were singly housed for the duration of the experiment. Animals in the cognitively characterized cohort were trained simultaneously. All young rats began training at 3 months of age. Aged rats began training at 23 and 25 months of age. Originally the cohorts were matched in size but 2 aged animals died before they could be tested and 2 aged animals failed to be trained on the task and were euthanized for biochemical analysis only. During habituation, training, and testing, animals were restricted to ~8-12 g per day of food to maintain motivation for the task. Animals were first habituated to the T-maze and rewards. Once the rat was acclimated to receiving rewards in the maze they were trained on forced alternation where the animal was forced to pick the opposite arm from their previous reward. Finally, animals were trained with a 0 sec delay to alternate between arms on the T-maze. Once they accomplished this training, they began their testing and were euthanized following the 20th day of testing.

Animals in the extreme age cohort were all handled at least 4 times prior to euthanasia to reduce stress but were otherwise were undisturbed and singly housed for at least 2 weeks. Young animals arrived at the facility at 3 months of age, were acclimated for about 2 weeks and then euthanized. To ensure consistency with aged animals, euthanasia was spread out over a few days and animals came from two separate cohorts. Aged animals were closely monitored for signs of health deterioration, at any sign of deteriorating health the animals were euthanized. As long as the animals stayed healthy, they were maintained until 30 months of age upon which they were euthanized. The minimum age was 24.5 months of age and 30% of animals reached the maximum 30 month time point. The distribution of ages is shown in Figure S2A.

**Protein Quantification**

Proteins were transferred onto a 2 μm nitrocellulose membrane and blocked in 5% milk. Primary antibodies were incubated at 4ºC overnight and in secondary antibody at room temperature for 1 hr. Antibodies were prepared in 50% Licor Odyssey blocking buffer/50% PBS-T (PBS with 0.1% Tween). Quantification of bands was done in ImageStudio Lite with background subtraction calculated by the average intensity immediately above and below the band(s) of interest. All markers are normalized by a loading control unless they were normalized within a single blot. Tau levels in brain lysates were calculated as the ratio of normalized pS214-tau over total tau. Each marker was normalized to a GAPDH loading control. In vitro samples were blotted for tau and pS214-tau with different species antibodies on the same blot and thuspS214-tau over total tau were directly normalized.

**Rat Primary Cortical Neuron Culture**

E19 embryos were removed from timed-pregnant Sprague-Dawley rats (Charles River). Meninges were removed and the cortical regions were dissected. Neurons were dissociated by mechanical dissociation with an 18G, then a 21G, needle following a 20 min 0.25% trypsin incubation. Dissociated neurons were filtered through a 0.40-μm nylon cell strainer. Cells were grown in 6 well plates coated with 0.1 mg/mL poly-D-lysine at a density of 600,000 cells per well. Cultures were grown in a Neurobasal media with 2% B27, 1% Glutamax, 1% Sodium Pyruvate, and 0.5% penicillin/streptomycin. A partial (40%) media change was made every 3-4 days.
