# Supplementary figures and images for: Phosphodiesterase PDE4D Is Decreased in Frontal Cortex of Aged Rats and Positively Correlated With Working Memory Performance and Inversely Correlated With PKA Phosphorylation of Tau
Source: Front Aging Neurosci. 2020 Oct 28;12:576723. doi: 10.3389/fnagi.2020.576723 (PMC7655962; doi:10.3389/fnagi.2020.576723)

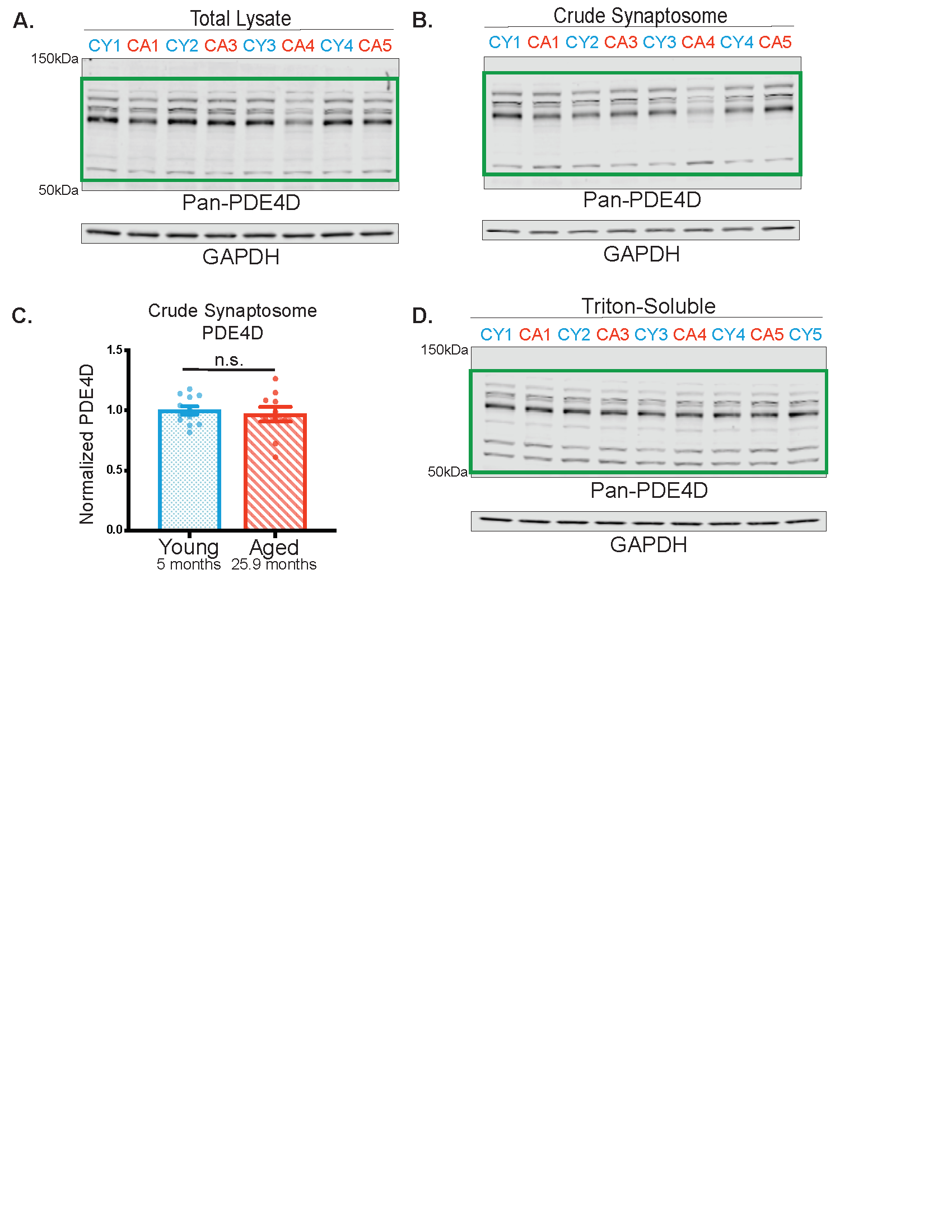

Supplement: Supplementary Figure 1 — PDE4D analysis in FC of cognitively characterized rats. (A) Representative blot of PDE4D in total sucrose lysate from the cognitively characterized cohort. All bands of PDE4D were utilized for quantification as illustrated by the quantified area outlined by the green rectangle. Lanes are labeled by their animal ID which is color-coded blue for young animals and red for aged animals. (B) Representative blot of PDE4D in the crude synaptosomal fraction from cognitively characterized animals. All bands of PDE4D were utilized for quantification as illustrated by the quantified area outlined by the green rectangle. (C) Crude synaptosomal PDE4D normalized by GAPDH was compared between young (blue, N = 10) and aged (red, N = 10) by an unpaired t-test (p = 0.6332). (D) Representative blot of PDE4D in triton-soluble lysate from the cognitively characterized cohort. Lanes are labeled by their animal ID which is color-coded blue for young animals and red for aged animals. All bands of PDE4D were utilized for quantification as illustrated by the quantified area outlined by the green rectangle. [file Image_1.TIFF]

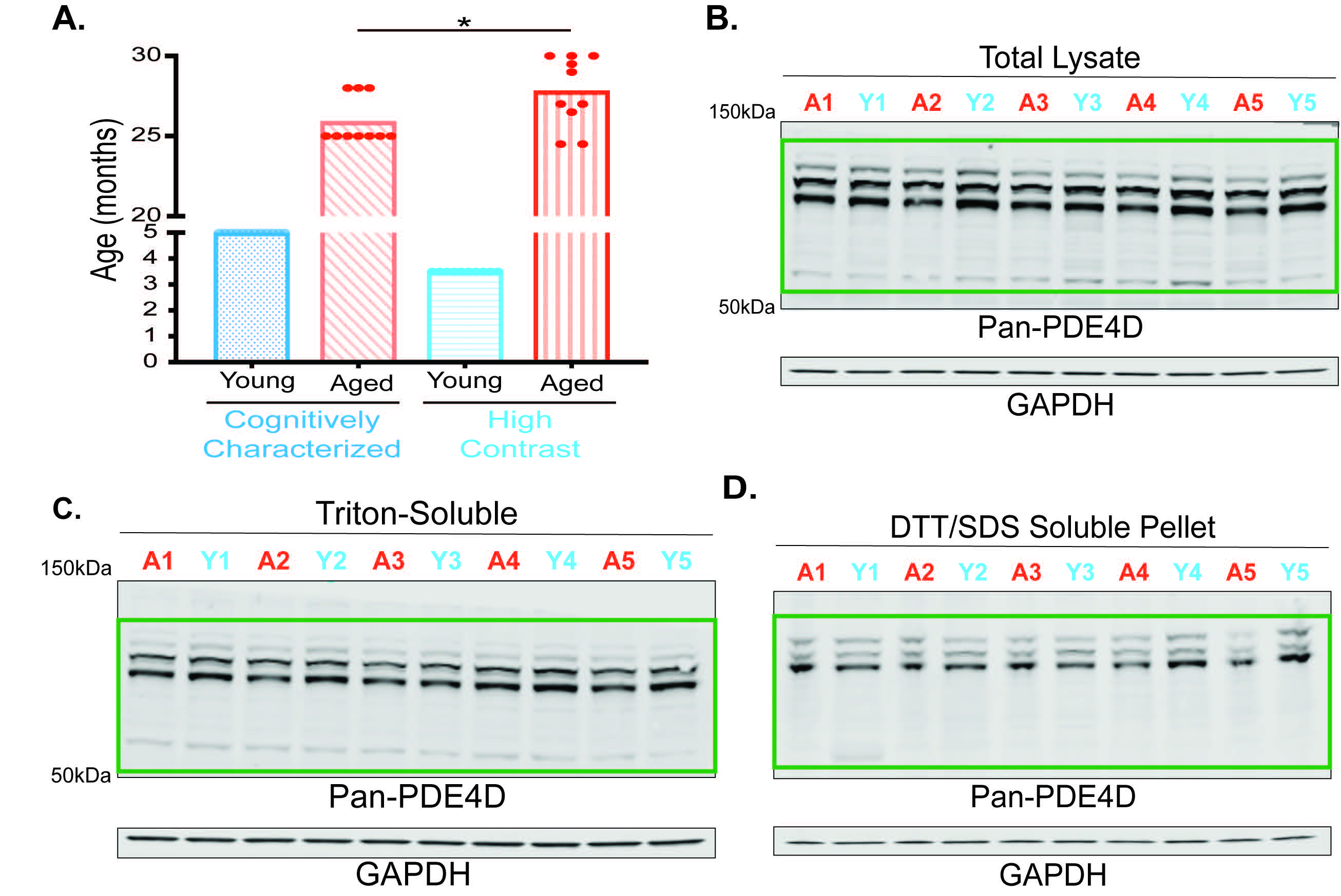

Supplement: Supplementary Figure 2 — PDE4D analysis in FC of an extreme aged cohort of rats. (A) Ages of the two rat cohorts analyzed in this study. Young animals are shown in blue and aged animals are shown in red. The two groups are distinguished by the pattern of the box plot. Cognitively characterized animals are shown by dots for young animals and diagonal lines for aged. Extreme aged animals are represented by horizontal lines for young animals and vertical lines for aged animals. Sidak’s multiple comparison test was used to compare the age difference between cognitively characterized and extreme aged animals. The two aged cohorts significantly differed in age (∗p = 0.016). (B) Representative blot of PDE4D in total lysate from the extreme age cohort. All bands of PDE4D were utilized for quantification as illustrated by the quantified area outlined by the green rectangle. The same molecular weight region was analyzed in all blots (B–D). Lanes are labeled by their animal ID which is color-coded blue for young animals and red for aged animals. (C) Representative blot of PDE4D in triton-soluble lysate from the extreme age cohort. Area of quantifications is highlighted by the green rectangle. Animals are labeled by their animal ID which is color-coded blue for young animals and red for aged animals. (D) Representative blot of PDE4D in the pellet resulting from the centrifugation of total lysate from the extreme age cohort. Area of quantifications is highlighted by the green rectangle. Lanes are labeled by their animal ID which is color-coded blue for young animals and red for aged animals. [file Image_2.TIFF]

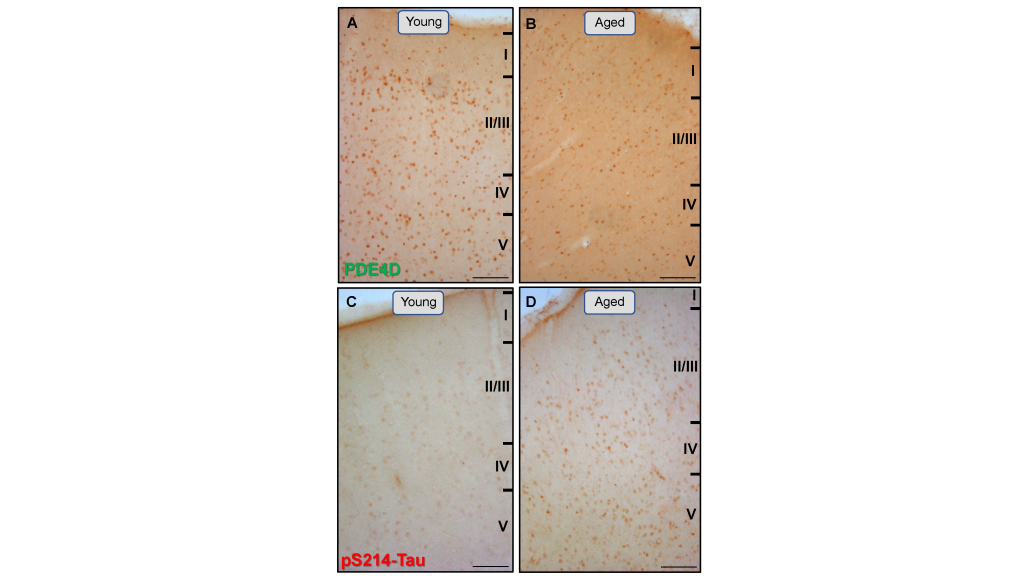

Supplement: Supplementary Figure 3 — PDE4D and pS214-tau immunohistochemistry across cortical layers in rat prelimbic mPFC. (A) Immunolabeling for PDE4D in young rat mPFC. Immunopositive cells are distributed throughout the cortical neuropil across all layers. (B) Immunolabeling for PDE4D in aged rat mPFC, with sparser labeling across all cortical layers. (C) Expression of pS214-tau in young rat mPFC with very few immunopositive cells. (D) Increased density and intensity of pS214-tau labeling across cortical layers in aged rat mPFC. Approximate depth of cortical layers is demarcated in each micrograph. Scale bars, 100 μm. [file Image_3.TIF]

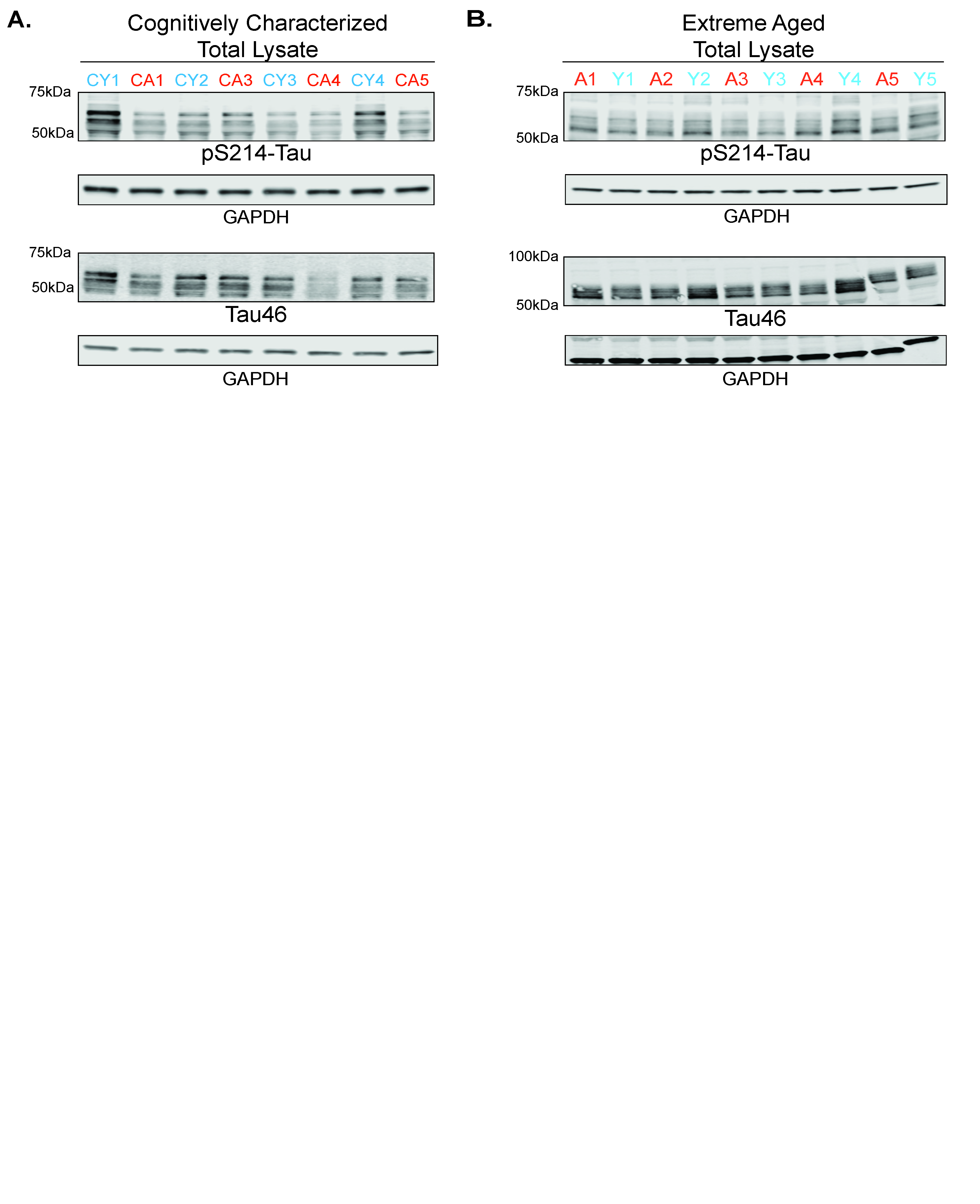

Supplement: Supplementary Figure 4 — pS214-tau in rat FC. (A) Representative blot of pS214-tau and total tau in total FC lysates from a cognitively characterized cohort. Lanes are labeled by their animal ID which is color-coded blue for young animals and red for aged animals. (B) Representative blot of pS214-tau and total tau in total lysate from an extreme aged cohort. Lanes are labeled by their animal ID which is color-coded blue for young animals and red for aged animals. [file Image_4.TIFF]
